# Genetic and Agronomic Analysis of Tobacco Genotypes Exhibiting Reduced Nicotine Accumulation Due to Induced Mutations in *Berberine Bridge Like* (*BBL*) Genes

**DOI:** 10.3389/fpls.2020.00368

**Published:** 2020-04-03

**Authors:** Ramsey S. Lewis, Katherine E. Drake-Stowe, Crystal Heim, Tyler Steede, William Smith, Ralph E. Dewey

**Affiliations:** Department of Crop and Soil Sciences, North Carolina State University, Raleigh, NC, United States

**Keywords:** tobacco, *Nicotiana tabacum*, nicotine, alkaloids, gene editing, regulation

## Abstract

Genetic methodologies for reducing nicotine accumulation in the tobacco plant (*Nicotiana tabacum* L.) are of interest because of potential future regulations that could mandate lowering of this alkaloid in conventional cigarettes. Inactivation of tobacco genes such as the *Berberine Bridge Like* (*BBL*) gene family believed to encode for enzymes involved in one of the latter steps of nicotine biosynthesis could be a viable strategy for producing new tobacco cultivars with ultra-low leaf nicotine accumulation. We introduced deleterious mutations generated via ethyl methanesulfonate treatment of seed or gene editing into six known members of the *BBL* gene family and assembled them in different combinations to assess their relative contribution to nicotine accumulation. Significant reductions (up to 17-fold) in percent leaf nicotine were observed in genotypes homozygous for combined mutations in *BBL-a*, *BBL-b*, and *BBL-c*. The addition of mutations in *BBL-d1*, *BBL-d2*, and *BBL-e* had no additional significant effect on lowering of nicotine levels in the genetic background studied. Reduced nicotine levels were associated with reductions in cured leaf yields (up to 29%) and cured leaf quality (up to 15%), evidence of physiological complexities within the tobacco plant related to the nicotine biosynthetic pathway. Further nicotine reductions were observed for a *BBL* mutant line cultivated under a modified production regime in which apical inflorescences were not removed, but at the expense of further yield reductions. Plants in which *BBL* mutations were combined with naturally occurring recessive alleles at the *Nic1* and *Nic2* loci exhibited further reductions in percent nicotine, but no plant produced immeasurable levels of this alkaloid. Findings may suggest the existence of a minor, alternative pathway for nicotine biosynthesis in *N. tabacum*. The described genetic materials may be of value for the manufacture of cigarettes with reduced nicotine levels and for future studies to better understand the molecular biology of alkaloid accumulation in tobacco.

## Introduction

Nicotine is a highly studied plant natural product produced in significant quantities by the species *Nicotiana tabacum* L., commonly known as tobacco, and numerous other members of the *Nicotiana* genus. This pyridine alkaloid is synthesized in tobacco roots and subsequently translocated to aerial plant parts in processes stimulated by plant wounding or loss of the apical inflorescence. Nicotine accumulation likely plays a role in natural plant defense against herbivores. Nicotine also plays an important role in human society as it is the primary addictive substance in manufactured tobacco products such as combustible cigarettes which have well-studied toxicant profiles. The United States Food and Drug Administration (FDA) lists 93 tobacco and tobacco smoke chemical constituents designated as “harmful and potentially harmful” due to their association with carcinogenesis, addiction, or respiratory, cardiovascular, reproductive, or developmental toxicity (United States Food and Drug Administration, 2012).

Nicotine *per se* is not recognized as a carcinogen, but the World Health Organization (2015) and the United States Food and Drug Administration (2018) have recommended a mandated lowering of nicotine levels in combustible cigarettes to non-addictive levels in order to reduce overall addiction to such products and to lower corresponding toxicant exposure. The specific concentrations at which nicotine becomes non-addictive in combustible cigarettes may be difficult to determine and may vary amongst individuals, but Benowitz and Henningfield (1994) have predicted tobacco filler nicotine contents of between 0.02 and 0.03% to be below a “sub-threshold level of addiction.” The World Health Organization (2015) has recommended lowering of nicotine content of the tobacco filler to below 0.04%. Percent nicotine on a dry weight basis in conventional tobacco cultivars typically ranges from between 1.0 and 5.0%, with observed variability being due to market type (burley, flue-cured, dark, cigar, or Oriental), plant genetics, growing environment, and stalk position. Manufacturers blend sourced cured leaf to produce cigarette tobacco filler with between 1.0 and 2.0% nicotine on a dry weight basis.

Various methods of chemical extraction have reportedly been used to achieve 80–98% reductions in nicotine content of tobacco filler (Seligman, 1983; Philip Morris United States, 1994; Coffa et al., 2016). Increased costs associated with chemical extraction, and the potential for co-extraction of compounds that positively affect organoleptic properties, make these approaches unattractive, however. Use of modified plant genetics is a preferred route to achieve reduced cigarette nicotine levels, if low-nicotine cultivars can be developed without substantially reducing cured leaf yields or otherwise undesirably altering chemical composition.

Genetic approaches to develop new tobacco cultivars with reduced potential for nicotine accumulation include the use of (1) genetic variability that naturally exists within *N. tabacum* or closely related species, (2) genetic variability induced by gene editing or mutagen treatment, or (3) novel variation generated via genetic engineering (reviewed by Lewis, 2018). Wide variation exists for alkaloid accumulation amongst diverse tobacco materials in the United States *Nicotiana* Germplasm Collection, ranging from 0.02 to 6.55% on a dry weight basis (Sisson and Saunders, 1982). The ultra-low nicotine levels of 0.04% recommended by the World Health Organization are not routinely observed amongst the lowest-alkaloid material, however. Recessive alleles at the *Nic1* and *Nic2* (also known as *A* and *B*) loci have been found to contribute to major reductions in nicotine and associated alkaloids (nornicotine, anabasine, and anatabine) from between 1.5 and 4.5% to approximately 0.20–0.45% (Legg and Collins, 1971; Lewis, 2018). This allelic variability is well known to be associated with reduced cured leaf yields and quality, however (Chaplin and Weeks, 1976).

Knowledge of specific genes identified to be involved in nicotine biosynthesis (Dewey and Xie, 2013) allows for the use of technologies such as RNA interference to lower their expression and achieve corresponding reductions in nicotine accumulation (Lewis, 2018). Commercialization of “GMO” tobacco cultivars is subject to variable levels of complicating regulatory oversight around the world, however. As an alternative to genetic engineering, gene silencing achieved via induced mutation or gene editing might be used to realize reductions in nicotine. Because of the polyploid nature of *N. tabacum*, mutations in multiple gene copies are often necessary to achieve a desired phenotype. The increased complexity of mutation breeding in a polyploid species can be offset by the fact that outcomes of such breeding approaches are not considered regulated articles in many parts of the world.

The *Berberine Bridge Like* (*BBL*) gene family was previously identified to encode for enzymes involved in one of the final steps of the *N. tabacum* nicotine biosynthetic pathway, although their precise role is currently not understood (Kajikawa et al., 2011). This family is comprised of six closely related members, three of which are expressed to significant degrees (*BBL-a*, *BBL-b*, and *BBL-c*) and three of which are lowly expressed (*BBL-d1*, *BBL-d2*, and *BBL-e*) (Kajikawa et al., 2017). The objectives of the current research were to establish nearly isogenic versions of elite flue-cured and burley tobacco cultivars that differed for the presence/absence of deleterious mutations in each of these six known *BBL* genes, and to evaluate these materials for altered alkaloid profiles and potential corresponding changes in yield and cured leaf quality. It was also of interest to determine if further reductions in nicotine could be achieved by (1) growing these modified tobaccos under a production regime where apical inflorescences are not removed, and (2) combining induced *BBL* mutations with the naturally occurring recessive alleles at the *Nic1* and *Nic2* loci.

## Materials and Methods

### Development and Testing of Nearly Isogenic Lines Carrying Mutations in BBL-a, BBL-b, and BBL-c

Treatment of tobacco seeds with ethyl methanesulfonate was previously used to introduce genome-wide mutations into burley tobacco breeding line DH98-325-6 (Lewis et al., 2010) and single nucleotide substitution mutations leading to truncated protein products were identified in the three most highly expressed *BBL* genes (*BBL-a*, *BBL-b*, and *BBL-c*) (Lewis et al., 2015) (Table 1). We subsequently used the backcross breeding method to transfer the three deleterious *BBL* mutations to tobacco cultivars K326, TN 90, and TN90 SRC. K326 is a popular flue-cured tobacco cultivar marketed as a fertile inbred line. TN 90 is a historically important burley tobacco cultivar also marketed as a fertile inbred line. TN 90 SRC (TN 90 **S**table **R**educed **C**onverter) is a nearly isogenic version of TN 90 that was produced by transferring deleterious mutations in three nicotine demethylase genes (*CYP82E4*, *CYP82E5*, and *CYP82E10*) (Lewis et al., 2010) to TN 90 using seven generations of backcrossing. These three gene mutations dramatically reduce the demethylation of nicotine to form nornicotine, a secondary alkaloid considered undesirable because of its association with the accumulation of the carcinogen *N*-nitrosonornicotine in cured tobacco leaves (Lewis et al., 2008).

**TABLE 1:** Deleterious mutations identified and studied for six *BBL* genes in tobacco.

Gene	% Amino acid Similarity to BBL-a	Mutation	Position from ATG	Amino acid Change
*BBL-a*	–	G/A	681	W227Stop
*BBL-b*	92.8	G/A	438	W146Stop
*BBL-c*	82.6	C/T	448	Q150Stop
*BBL-d1*	64.0	Indel (−106, +11 bp)	163	Frameshift mutation
*BBL-d2*	65.4	Indel (−2, +19 bp)	271	Frameshift mutation
*BBL-e*	85.4	Indel (−19, +11 bp)	263	Frameshift mutation

K326, TN 90, and TN 90 SRC were each initially hybridized with an individual plant in which the *BBL-a*, *BBL-b*, and *BBL-c* truncation mutations were initially assembled in triple homozygous conditions (Lewis et al., 2015). Each F_1_ was then backcrossed to their respective recurrent parents, and selection for all three mutations was carried out using Kompetitive Allele Specific PCR (KASP) markers. The backcross breeding method was carried out to the BC_7_F_1_ generation for each of the three lines, at which time triple heterozygous mutant genotypes were self-pollinated to produce the BC_7_F_2_ generation. BC_7_F_2_ individuals were genotyped to identify all eight possible homozygous genetic combinations (Supplementary Table 1), which were then self-pollinated to produce BC_7_F_3_ seed of the nearly isogenic lines (NILs) for each genetic background. Numerical nomenclature is hereafter used to refer to each of these genotypes, where a “0” indicates the homozygous “wild-type” condition at a particular locus, “2” designates the homozygous mutant condition at a particular locus, and “1” indicates a heterozygote (Supplementary Table 1). For example, the designation “K326 (210)” indicates a line homozygous for the mutant genotype at the *BBL-a* locus, heterozygous for the mutant genotype at the *BBL-b* locus, and homozygous for the wild type allele at the *BBL-c* locus.

The eight K326-derived NILs were evaluated for yield, physical cured leaf quality, and cured leaf chemistry in comparison with K326, NC95, and corresponding NC95 isolines MAFC5 (*Nic1*/*Nic1 nic2*/*nic2*) (Chaplin, 1986), LMAFC34 (*nic1*/*nic1 Nic2*/*Nic2*) (Chaplin and Burk, 1984), and LAFC53 (*nic1*/*nic1 nic2*/*nic2*) (Chaplin, 1975). An F_1_ hybrid between K326 and K326 (222), designated as K326 (111) was also included. Experiments were carried out at three North Carolina field locations (Upper Coastal Plain Research Station, Rocky Mount; Cunningham Research Station, Kinston; and the Oxford Tobacco Research Station, Oxford) during 2016 and 2017, for a total of six field environments. Experimental units at each location consisted of single 20-plant rows managed according to standard flue-cured production practices for North Carolina. Intra-row spacing was 56 cm at all three locations, while inter-row spacing was 122 cm at the Oxford and Rocky Mount locations and 112 cm at the Kinston locations.

To investigate the level of leaf green color immediately prior to harvest of the top leaves (an indicator of leaf ripening), the chlorophyll contents of the upper most leaves of K326, K326 (222), NC95, MAFC5, LMAFC34, and LAFC53 were estimated using a handheld Konica Minolta SPAD-502 meter (Konica Minolta, Tokyo, Japan) according to manufacturer’s instructions. One measurement was taken for the top leaf of each of 10 plants per plot and averages were calculated for each plot.

Leaves were harvested in four separate harvests (primings) and flue-cured. Each priming was weighed to generate yield data, and official USDA quality grades were assigned by a former USDA grader. Cured leaf quality was also assessed using the 2017 North Carolina Flue-Cured Tobacco Grade Index (North Carolina Cooperative Extension Service, 2017). Value per hundred weight ($ cwt^–1^) and value ($) ha^–1^were calculated based on the 2017 flue-cured tobacco price index (North Carolina Cooperative Extension Service, 2017). Plot values for grade index and $ cwt^–1^ were calculated using a weighted average over all four primings. Fifty-gram de-stemmed cured leaf samples were prepared for each plot by compositing cured leaf from each priming on a weighted-mean basis. Oven-dried samples were ground to pass through a 1-mm sieve and analyzed for alkaloid profiles (expressed as a percentage of dry weight) as previously outlined by Lewis et al. (2015). Percent reducing sugars were analyzed according to the method of Davis (1976).

The eight TN 90-derived and eight TN 90 SRC-derived *BBL* mutant NILs lines were evaluated at two locations representative of burley tobacco producing environments in North Carolina during the 2016 and 2017 growing seasons: the Mountain Research Station at Waynesville, and the Upper Mountain Research Station at Laurel Springs. Materials were evaluated in comparison with TN 90, TN 90 SRC, Burley 21, and the corresponding Burley 21 isolines HI Burley 21 (*Nic1*/*Nic1 nic2*/*nic2*), LI Burley 21 (*nic1*/*nic1 Nic2*/*Nic2*), and LA Burley 21 (*nic1*/*nic1 nic2*/*nic2*) (Legg et al., 1970). Experimental units were single 20 plant rows managed according to suggested production practices for burley tobacco production in North Carolina. Row and plant spacing were 122 cm and 46 cm, respectively. To investigate the level of leaf green color immediately prior to harvest, the chlorophyll contents of the upper most leaves of TN 90, TN 90 SRC, TN 90 (222), TN 90 SRC (222), Burley 21, HI Burley 21, LI Burley 21, and LA Burley 21 were estimated using a SPAD-502 meter. The top of leaf of each of 10 plants per plot was measured, and averages were calculated for each plot.

Plots were stalk cut at maturity, speared onto sticks, and air-cured in structures protected from rainfall. After curing, leaves were stripped into four stalk positions, weighed, and assigned a USDA grade by a former USDA tobacco grader. A grade index was also assigned using the index of Bowman et al. (1989). Plot values for grade index were calculated using a weighted average over all four stalk positions. Fifty-gram cured leaf samples were prepared for each plot, processed, and analyzed for alkaloid profiles as previously described.

### Development and Testing of Nearly Isogenic Lines Carrying Mutations in BBL-d1, BBL-d2, and BBL-e

To investigate the potential role of three additional, but lowly expressed, members of the *BBL* gene family in nicotine biosynthesis (Kajikawa et al., 2017), gene editing was used to introduce deleterious mutations into *BBL-d1*, *BBL-d2*, and *BBL-e* (Table 1). Two proprietary ARCUS sequence-specific nuclease (Precision Biosciences, Durham, NC, United States) were strategically designed to introduce mutations into the three minor *BBL* genes, one specific for *BBL-e* and the other specific for *BBL-d1* and *-d2*. TN90 SRC (222) was a source of tobacco leaf explants used in *Agrobacterium*-based transformation to introduce the transgene construct carrying the ARCUS nuclease gene. Transgenic plants identified as possessing mutations in the three minor genes were self-pollinated and, after selection against plants carrying transgene insertions, all possible homozygous mutant combinations were identified in the TN90 SRC (222) genetic background. Plants were self-pollinated to produce stable lines that were designated using the previously described nomenclature (Supplementary Table 1). These materials were included in the 2017 burley tobacco field experiments (two environments) and evaluated as previously described.

### Testing of Low Alkaloid Flue-Cured Tobacco Genotypes Under Topped and Non-topped Production Regimes

K326 and NC95 were evaluated, along with their respective low-nicotine NILs K326 (222) and LAFC53 (*nic1*/*nic1 nic2*/*nic2*), in a 2018 field experiment to investigate the extent to which nicotine levels might be further reduced by growing tobacco lines with a reduced genetic potential for alkaloid accumulation under a non-conventional production regime where plants are not topped (i.e., the apical inflorescence is not removed). This experiment was carried out in a field environment near Clayton, NC, using recommended tobacco production methods for the region (with the exception of non-topping). The experimental design was a split plot design with four replications. The main plot factor was production regime (topped versus non-topped), while the subplot factor was genotype. Experimental units were five plant rows with intra-row plant spacing and row spacing of 56 and 112 cm, respectively. All plants within the topped portion of the experiment were topped on the same day after greater than 95% of the plants flowered. Plants were generally topped 1–2 leaves below the lowest flowering branch, where the smallest remaining leaves were at least 20 cm in length and 10 cm in width. Immediately post-topping, each plant was treated with a downstalk application of Prime + EC (Syngenta Crop Protection, Greensboro, NC, United States) according to manufacturer’s instructions in order to suppress lateral meristem development (suckering). Chemical sucker control was supplemented with hand removal, when necessary.

Plots were harvested in four primings, according to the rate of ripening, from the bottom to the top of the stalk. Four to six leaves were harvested in each priming. Per plot fresh leaf weights were recorded in the field and converted to kg ha^–1^ fresh weight yields. Five leaves from each priming for each plot were collected, oven dried, and analyzed for individual stalk position alkaloid profiles as previously described. Alkaloid percentages for a composite sample for each plot were calculated based on the relative weights of each priming.

### Combining BBL Mutations With Recessive Alleles at the Nic1 and Nic2 Loci

It was of interest to determine whether nicotine accumulation of K326 (222) could be further lowered by incorporating the recessive alleles at the *Nic1* or *Nic2* loci into this genetic background. K326 (222) was initially hybridized with LAFC53 (*nic1*/*nic1 nic2*/*nic2*). BC_2_F_1_ progeny were subsequently developed after backcrosses to K326 (222) accompanied with selection for the homozygous mutant condition at the *BBL-a*, *BBL-b*, and *BBL-c* loci using the previously described KASP markers and selection for the recessive *nic1* and *nic2* alleles using SNP markers described by Adams et al. (2016). *bbl-a*/*bbl-a bbl-b*/*bbl-b bbl-c*/*bbl-c Nic1*/*nic1 Nic2*/*nic2* BC_2_F_1_ individuals were self-pollinated and triple homozygous *bbl* mutant BC_2_F_2_ plants were identified via genotyping that were either *Nic1*/*Nic1 nic2*/*nic2*, *nic1*/*nic1 Nic2*/*Nic2*, or *nic1*/*nic1 nic2*/*nic2*. Such BC_2_F_2_ plants were self-pollinated to produce BC_2_F_3_ families that were evaluated in comparison with K326, K326 (222), and LAFC53 for nicotine accumulation in a single 2019 field environment near Clayton, NC. Plants were managed according to standard flue-cured production practices for North Carolina. The experimental design was a completely randomized design with each genotype being represented by between 12 and 37 plants. The top two leaves of each plant were harvested 21 days after topping, air cured, and analyzed for alkaloid profiles as previously described.

### Data Analysis

Analyses of variance appropriate for analyzing randomized complete block designs or split plot designs were carried out for all measured traits using PROC MIXED of SAS 9.3 (SAS Institute, Cary, NC, United States) according to Littell et al. (1996). Environments were considered as random effects, while genotype and production regime (topped versus nontopped) were considered as fixed effects. Means separations were performed via appropriate F-tests or by using Fisher’s least significant difference test (α = 0.05).

## Results

### Evaluation of K326 Flue-Cured Tobacco Isolines Containing Mutations in BBL-a, BBL-b, and BBL-c

**FIGURE 1:**
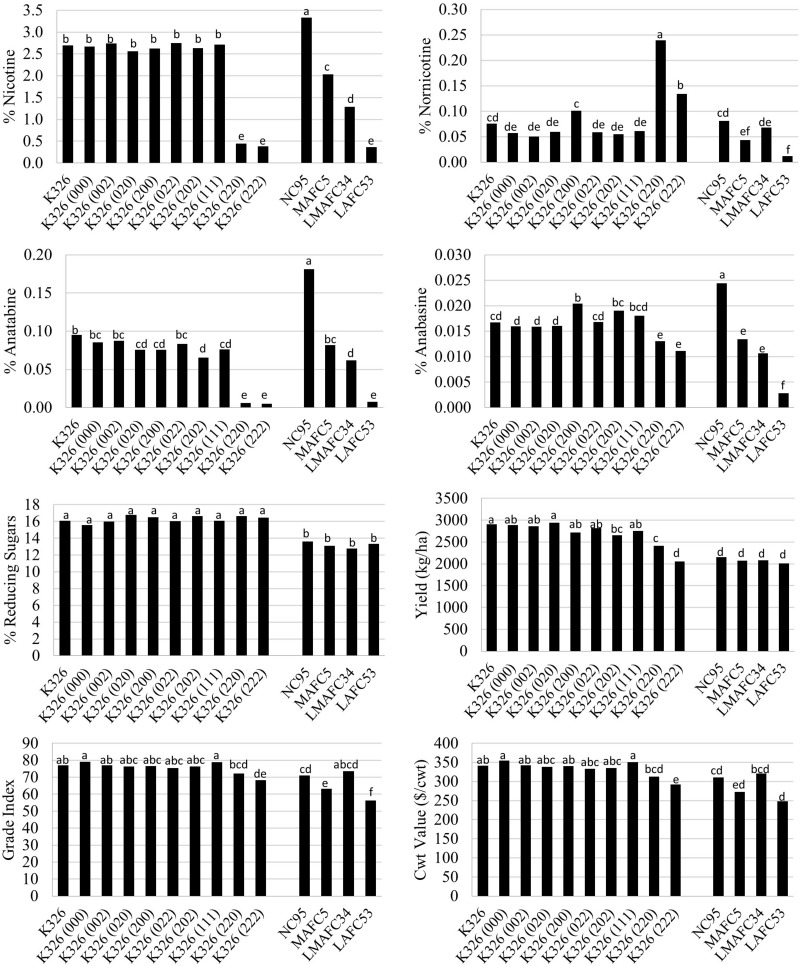
Entry means for flue-cured genetic materials evaluated for alkaloid levels, yield, and quality determinations. Means are averaged over six 2016 and 2017 North Carolina field environments. Means with different letters are significantly different from each other at the *P* < 0.05 level of significance.

Evaluation of eight K326 homozygous *BBL* mutant lines and corresponding controls in six field environments indicated that that the addition of no single *BBL* gene mutation had a significant effect to lower nicotine accumulation (Figure 1). Only genotypes with mutations in both *BBL-a* and *BBL-b* exhibited significant (*P* < 0.05) reductions in nicotine as compared to K326. The K326 (220) and K326 (222) mutant lines accumulated 0.45 and 0.38% nicotine, respectively, as compared to 2.69% for K326. Progressive decreases in nicotine were observed as the number of recessive alleles at the *nic1* and *nic2* loci increased in the genetic background of NC95. Nicotine levels in the triple homozygous mutant line K326 (222) were not lower than that for LAFC53, the *nic1*/*nic1 nic2*/*nic2* isoline of flue-cured tobacco cultivar NC95.

Some small significant (*P* < 0.05) reductions were observed for percent anatabine in single mutant lines, but not for anabasine (Figure 1). Extreme reductions in percent anatabine were measured for K326 (220) and K326 (222). More modest, but still significant, reductions were found for percent anabasine for these two genotypes. Similar to nicotine, progressive decreases in anatabine and anabasine accumulation were found with the addition of recessive alleles at the *nic1* and *nic2* loci in an NC95 genetic background. Significant (*P* < 0.05) increases in percent nornicotine were observed for the double mutant genotypes K326 (220) and K326 (222) (Figure 1). No significant changes in percent reducing sugars were found amongst the K326 isolines or amongst the NC95 isolines.

**FIGURE 2:**
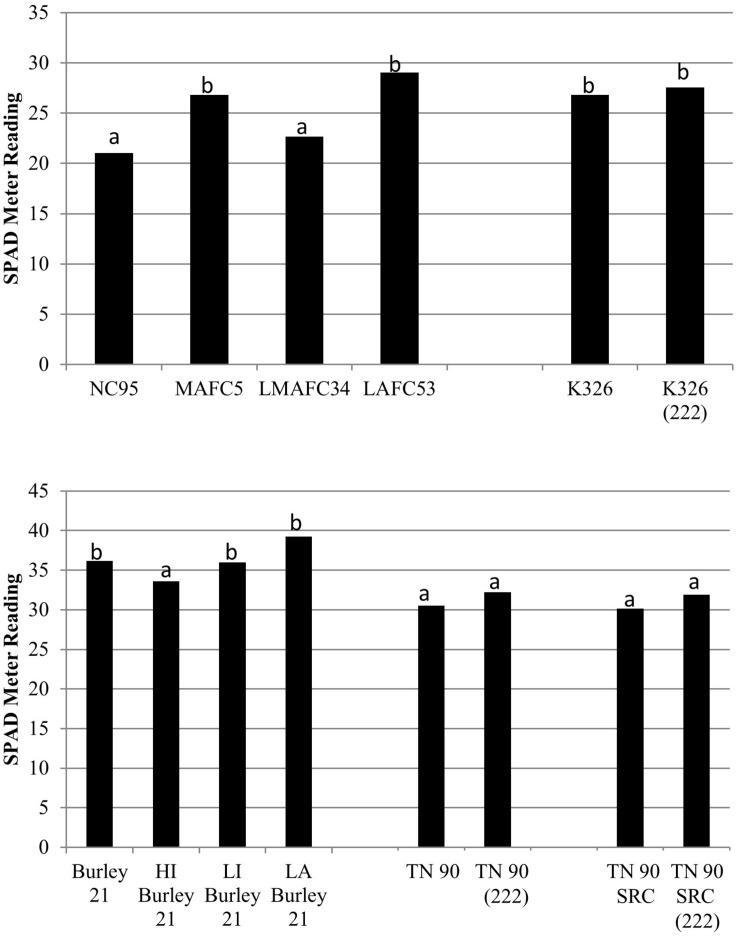
SPAD meter ratings for low alkaloid genetic materials and controls. Means are averaged over two 2017 North Carolina environments. Means with different letters are significantly different from each other at the *P* < 0.05 level of significance.

Prior to harvest of the last priming, the difference in leaf green color (as measured by a SPAD meter) between K326 and K326 (222) was found to be non-significant (*P* > 0.05) (Figure 2). In comparison, the NC95 isolines MAFC5 (*Nic1*/*Nic1 nic2*/*nic2*) and LAFC53 (*nic1*/*nic1 nic2*/*nic2*) were found to be significantly more green than NC95.

Significant observed reductions in percent nicotine for K326 (220) and K326 (222) were associated with significantly lower (*P* < 0.05) cured leaf yields (17.0 and 29.5% reductions, respectively) (Figure 1). Significant reductions in cured leaf quality also accompanied lower nicotine levels in K326 (222) as reflected by significantly lower grade index and cwt value. These characters were reduced by 11.3 and 14.5% relative to K326, respectively. No significant differences in yield were detected amongst the NC95 isolines, but cured leaf quality of MAFC5 and LAFC53 were significantly reduced relative to NC95. Cured leaf quality of the low nicotine mutant genotype K326 (222) was significantly better than that for LAFC53.

### Evaluation of TN 90 Burley Tobacco Isolines Containing Mutations in BBL-a, BBL-b, and BBL-c

**FIGURE 3:**
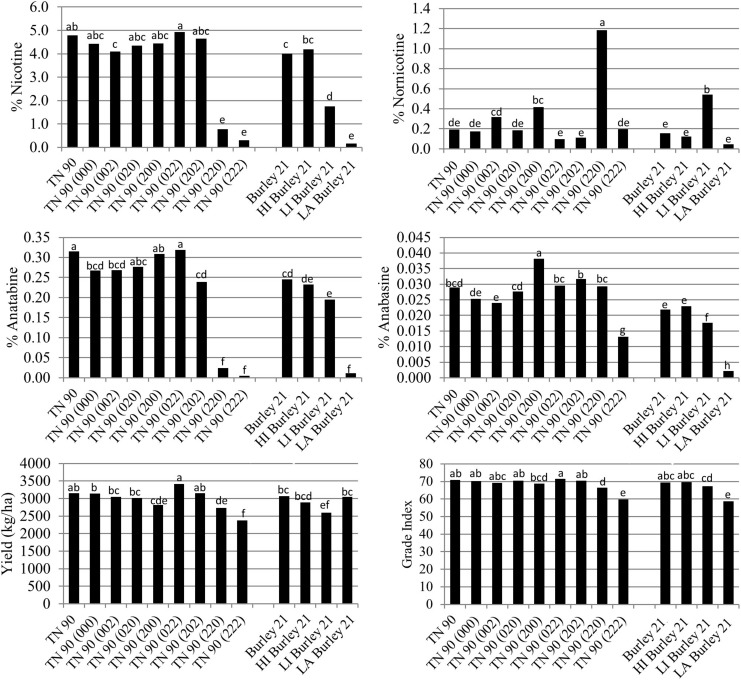
Entry means for TN90 burley tobacco genetic materials evaluated for alkaloid levels, yield, and quality determinations. Means are averaged over four 2016 and 2017 North Carolina field environments. Means with different letters are significantly different from each other at the *P* < 0.05 level of significance.

The *BBL-a*, *BBL-b*, and *BBL-c* mutations were also evaluated in all possible homozygous combinations in the genetic background of burley tobacco cultivar TN 90 in a total of four field environments. Relative to TN 90, the only TN 90 single mutant line exhibiting a slight significant (*P* < 0.05) reduction in percent nicotine was TN 90 (002) (Figure 3). Similar to the K326 genetic background, dramatic reductions in nicotine were only realized in double mutant genotypes TN 90 (220) and TN 90 (222). Significant (*P* < 0.05) differences were detected between the *nic1*/*nic2* isolines of burley tobacco cultivar Burley 21, with LI Burley 21 and LA Burley 21 exhibiting the second-lowest and lowest nicotine levels of the group, respectively (Figure 3). Percent nicotine for TN 90 (222) was not lower than that for LA Burley 21.

Relative to TN 90, small significant (*P* < 0.05) reductions in anatabine were detected for single mutant line TN 90 (002) and double mutant line TN 90 (202) (Figure 2). Dramatic reductions in anatabine were observed only for the double mutant genotypes TN 90 (220) and triple mutant TN 90 (222). Progressive decreases in percent anatabine were measured as the number of recessive alleles at the *nic1* and *nic2* loci increased in the Burley 21 genetic background. Relative to TN 90, a small significant reduction in anabasine content was observed for single *BBL* mutant line TN 90 (002), and a significant modest increase was measured for single *BBL* mutant line TN 90 (200). A significant modest decrease in percent anabasine was measured for the triple homozygous mutant TN 90 (222). Nornicotine accumulation was found to be significantly greater, relative to TN 90, in mutant lines TN 90 (200) and TN 90 (220).

Prior to stalk harvesting of burley tobacco plots, the degree of leaf greenness (as measured by a SPAD meter) between TN 90 and TN 90 (222) was found to be non-significant (*P* > 0.05) (Figure 2). In comparison, only the Burley 21 isoline HI Burley 21 (*Nic1*/*Nic1 nic2*/*nic2*) was found to be significantly less green than Burley 21. LA Burley 21 (*nic1*/*nic1 nic2*/*nic2*) exhibited the greatest average SPAD meter rating, but this was not significantly greater than that for Burley 21.

Nicotine reductions in double and triple homozygous mutant lines TN 90 (220) and TN 90 (222) were associated with significant (*P* < 0.05) reductions in cured leaf yields of 13.2 and 24.5%, respectively (Figure 3). Reductions in nicotine for these mutant genotypes was also found to be associated with significant (*P* < 0.05) reductions in cured leaf quality (as reflected by grade index) of 6.2 and 15.5%, respectively. Cured leaf quality of the low percent nicotine genotype TN 90 (222) was not significantly different than that for LA Burley 21, the *nic1*/*nic1 nic2*/*nic2* isoline of Burley 21.

### Evaluation of TN 90 SRC Burley Tobacco Isolines Containing Mutations in BBL-a, BBL-b, and BBL-c

**FIGURE 4:**
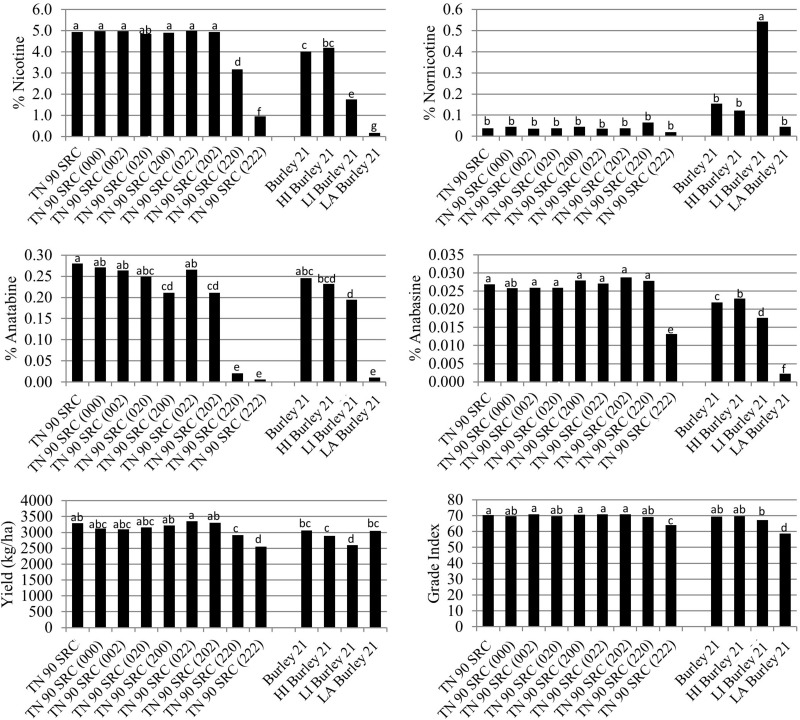
Entry means for TN90 SRC burley tobacco genetic materials evaluated for alkaloid levels, yield, and quality determinations. Means are averaged over four 2016 and 2017 North Carolina field environments. Means with different letters are significantly different from each other at the *P* < 0.05 level of significance.

The *BBL-a*, *BBL-b*, and *BBL-c* mutations were also evaluated in all possible homozygous combinations in a TN 90 SRC genetic background. As mentioned previously, the TN 90 SRC genotype differs from TN 90 in that TN 90 SRC carries deleterious mutations in each of three nicotine demethylase genes (Lewis et al., 2010), a genetic combination that dramatically reduces the demethylation of nicotine to form nornicotine. Relative to TN 90 SRC, percent nicotine was only significantly (*P* < 0.05) reduced in TN 90 SRC (220) and TN 90 SRC (222) (Figure 4).

Similar to the other genetic backgrounds, percent anatabine was dramatically and significantly (*P* < 0.05) reduced in the double and triple homozygous mutant genotypes TN 90 SRC (220) and TN 90 SRC (222), relative to TN 90 SRC (Figure 4). Slight significant reductions in percent anatabine were measured for TN 90 SRC (200) and TN 90 SRC (202). Relative to TN 90 SRC, the only mutant isoline exhibiting significantly (*P* < 0.05) lower anabasine accumulation was the triple homozygous mutant line, TN 90 SRC (222). No significant differences were observed between the TN 90 SRC mutant isolines for the accumulation of nornicotine (Figure 4).

The green color of TN90 SRC (222) was not significantly greater than that for TN 90 SRC immediately prior to stalk harvest (Figure 2). Nicotine reductions in double and triple homozogyous mutant lines TN 90 SRC (220) and TN 90 SRC (222) were accompanied by significant (*P* < 0.05) reductions in cured leaf yields of 11.7 and 22.5%, respectively (Figure 4). Cured leaf quality as measured by grade index was also significantly (*P* < 0.05) reduced by 8.7% in the triple homozygous mutant genotype as compared to TN 90 SRC.

### Evaluation of TN 90 SRC Burley Tobacco Isolines Possessing Mutations in up to Six BBL Genes

**FIGURE 5:**
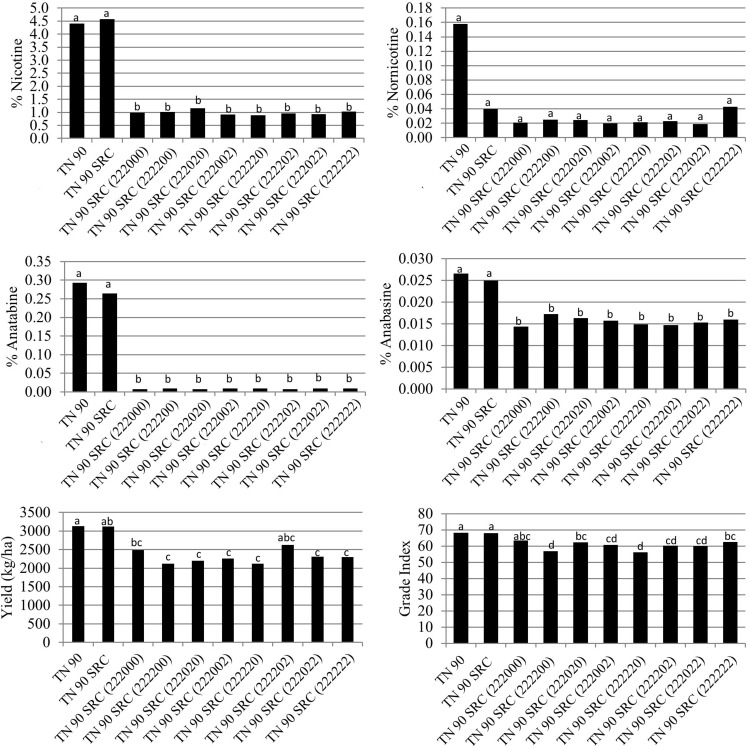
Entry means for TN 90 SRC burley tobacco genetic materials evaluated for alkaloid levels, yield, and quality determinations. Means are averaged over two 2017 North Carolina field environments. Means with different letters are significantly different from each other at the *P* < 0.05 level of significance.

Relative to TN 90 SRC (222), no additional significant changes in the accumulation of nicotine, nornnicotine, anatabine, or anabasine were found in genotypes in which additional *BBL* mutations (*bbl-d1*, *bbl-d2*, or *bbl-e*) were added to the genetic background of TN 90 SRC (222) (Figure 5). Yield was numerically lower in most materials containing at least four mutations, although these reductions were not statistically significant. Cured leaf quality as measured by grade index was significantly lower for two genotypes carrying at least four *BBL* mutations as compared to TN 90 SRC (222) (Figure 5).

### Testing of Low Alkaloid Flue-Cured Tobacco Genotypes Under Topped and Non-topped Production Regimes

**FIGURE 6:**
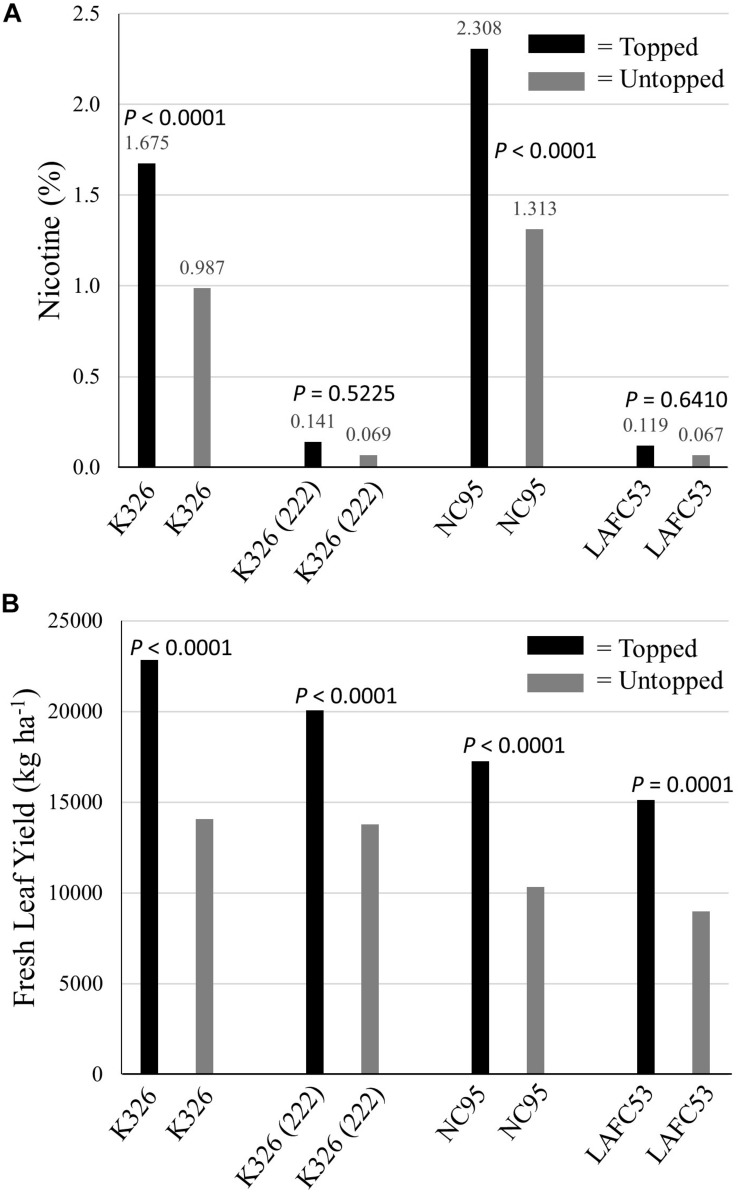
Percent nicotine **(A)** and fresh leaf yields **(B)** for four tobacco genotypes evaluated under topped and untopped production regimes. *P*-values for testing the level significance between two means within a genotype are indicated above the bars.

K326 and NC95 were evaluated in comparison with their low-alkaloid NILs K326 (222) and LAFC53 (*nic1*/*nic1 nic2*/*nic2*) under a conventional production regime (where all plants were topped at flowering) and under a non-conventional production regime in which flowers were not removed. Averaged over all tested genotypes, the untopped regime resulted in significantly lower (*P* = 0.0038) lower nicotine levels (averaged over all stalk positions) as compared to the production regime in which all plants were topped (Figure 6). Untopped K326 and NC95 exhibited significantly lower (*P* < 0.0001) percent nicotine (averaged over all stalk positions) relative to topped K326 and NC95, respectively (Figure 6). Although untopped K326 (222) and LAFC53 exhibited numerically lower percent nicotine for composite samples relative to topped K326 (222) and LAFC53, these differences were not statistically significant (*P* > 0.05). The lowest numerical average percent nicotine was measured for untopped LAFC53 (0.067%).

Averaged over all genotypes, green leaf yields of untopped tobacco were 37.4% lower than green leaf yields of topped tobacco (significantly different at *P* = 0.0015). Each of the four tested genotypes was significantly lower yielding (*P* < 0.0002) under the untopped versus the topped production regime (Figure 6).

**FIGURE 7:**
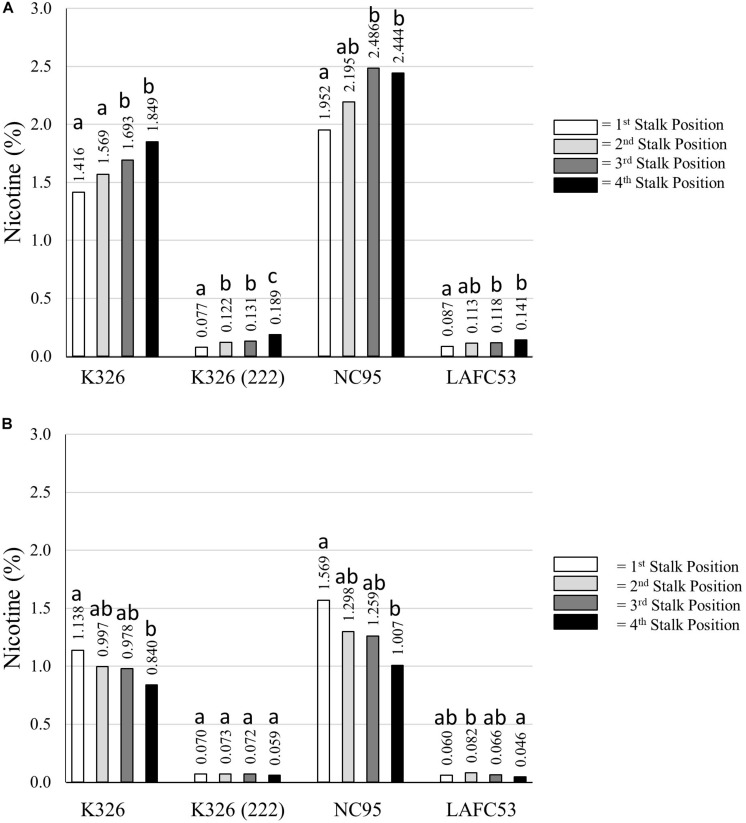
Percent nicotine for four different stalk positions for each of four different tobacco genotypes evaluated under topped **(A)** and untopped **(B)** production regimes. Means with different letters within a genotype are significantly different from each other at the *P* < 0.05 level of significance.

Average percent nicotine for each individual stalk position for each of the four tested genotypes is presented in Figure 7. For the topped tobacco entries, the general trend was one of increasing nicotine accumulation as the stalk position increased from lowest to highest. The lowest percent nicotine for topped tobacco (0.077%) was observed for the lowest stalk position for genotype K326 (222). For untopped tobacco, the trend was one of decreasing nicotine content as the stalk position increased for K326 and NC95 (Figure 7). These trends were not obvious and the range in nicotine levels between stalk positions was substantially less for the low alkaloid genotypes K326 (222) and LAFC53. The lowest average measured percent nicotine in the experiment was for the uppermost stalk position for untopped LAFC53 (0.046%).

### Combining BBL Mutations With Recessive Alleles at the Nic1 and Nic2 Loci

**FIGURE 8:**
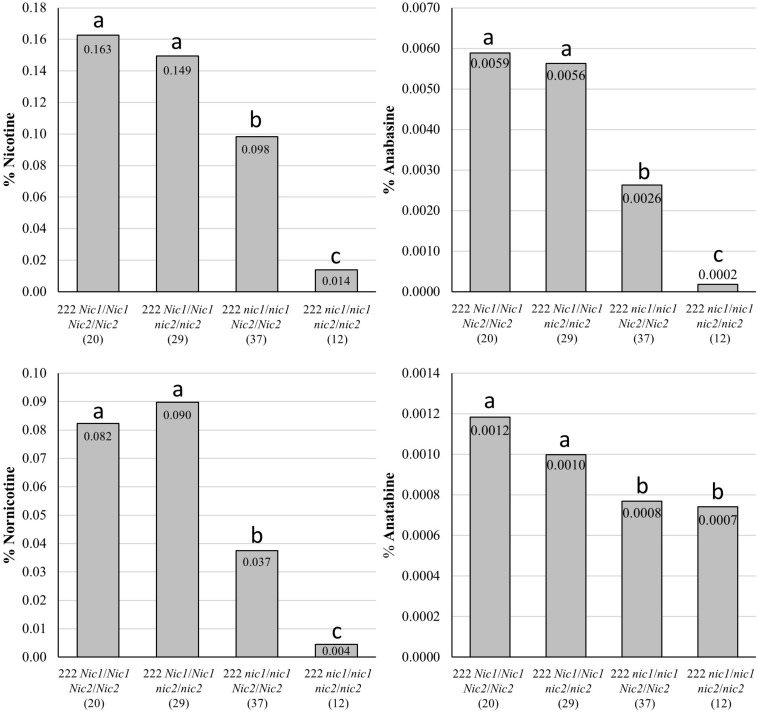
Mean alkaloid contents on a percent dry weight basis for top two leaves of BC_2_F_3_ individual plants fixed for mutations in *BBL-a*, *BBL-b*, and *BBL-c*, but segregating for recessive alleles at the *Nic1* and *Nic2* loci. Samples were collected 21 days after topping. Number of plants in each genotypic class is indicated within parenthesis. Genotypic means are indicated on individual bars, and means with the same letters are not significantly different from each other at the *P* > 0.05 level of significance as determined by *t*-tests.

Genotypes of the *bbl-a*/*bbl-a bbl-b*/*bbl-b bbl-c*/*bbl-c* genetic combination generally exhibited lower, but not significantly lower, alkaloid levels (with the exception of nornicotine) when combined with the recessive alleles at only the *Nic2* locus in a K326 genetic background (Figure 8). Significant reductions (*P* < 0.05) reductions in all alkaloids (*P* < 0.05) were observed for *bbl-a*/*bbl-a bbl-b*/*bbl-b bbl-c*/*bbl-c* individuals also carrying the recessive alleles at only the *Nic1* locus. The lowest alkaloid levels were measured for *bbl-a*/*bbl-a bbl-b*/*bbl-b bbl-c*/*bbl-c* plants that were also homozygous for the recessive alleles at both the *Nic1* and *Nic2* loci (Figure 8). The average nicotine content of plants with this genotype was very low (0.014%).

## Discussion

The potential for mandated lowering of nicotine levels in conventional cigarettes by regulatory agencies has caused a growing interest in genetic methodologies that might be used to reduce levels of this alkaloid in the tobacco plant. Ultra-low levels of nicotine (0.04% or below) for cigarette tobacco filler are currently being recommended, but there are no commercially available or publicly disclosed tobacco cultivars of any market type that routinely produce cured leaf with nicotine content this low under conventional production regimes (Lewis, 2018).

Extensive knowledge currently exists regarding enzymes directly involved in nicotine biosynthesis (Dewey and Xie, 2013). It might therefore seem straightforward to interfere with expression of associated genes to reduce nicotine to zero or close to zero. Very recently, Schachtsiek and Stehle (2019) reported on “nicotine-free tobacco” developed using CRISPR-Cas9-based gene editing of members of the *BBL* gene family. However, this work was carried out using poorly described leaf samples from non-topped plants of an unknown tobacco cultivar that were grown in pots in a greenhouse. In addition, this prior research did not determine the relative importance of the six known *BBL* genes on alkaloid accumulation and rigorous evaluations of corresponding effects on tobacco yield and quality were not carried out. Our results demonstrate how conclusions drawn from research related to tobacco alkaloid accumulation can differ according to whether the plants were evaluated in greenhouse versus field environments. Indeed, as shown here, greenhouse experiments can lead to misleading information for the tobacco regulatory and manufacturing communities.

Thus far, there are no published reports where silencing of a single target gene family has resulted in a nicotine-free tobacco genotype when grown under field conditions and conventional production regimes. In research described here, we assembled all possible homozygous combinations of truncation mutations in the most highly expressed members of the *BBL* gene family involved in one of the later steps of nicotine biosynthesis. The lowest-nicotine *BBL* mutant combinations resulted in reductions of between 5.8 and 17-fold. Averaged over all stalk positions, the lowest-nicotine *BBL* mutant combination exhibited 0.297% nicotine in the cured leaf. Dramatic reductions in nicotine accumulation were only observed in materials homozygous for mutations in the two most highly expressed *BBL* genes, *BBL-a* and *BBL-b*. Mutations in *BBL-c* resulted in only slight additional reductions in nicotine when combined with mutations in *BBL-a* and *BBL-b*. Mutations in *BBL-d1*, *BBL-d2*, and *BBL-e* appeared to have no significant effect on nicotine levels, either alone, or in combination with other mutations in the TN 90 SRC genetic background that was studied. In no genetic background did the lowest nicotine *BBL* mutant combination exhibit percent nicotine that was numerically lower than that for the conventionally developed low-alkaloid *nic1*/*nic1 nic2*/*nic2* breeding lines LA Burley 21 and LAFC53. Even the lowest nicotine stalk position of the best mutant combination was not beneath the recommended target nicotine level of 0.04%.

A typical objective of metabolic engineering is to achieve a desired chemical change without otherwise affecting other chemical attributes, growing efficiency, or derived product quality. Prior research has revealed great difficulty in using modified plant genetics to reduce nicotine levels without altering metabolic profiles or negatively affecting tobacco cured leaf yields or quality (Chaplin and Weeks, 1976; Kudithipudi et al., 2017). Similar to the *nic1*/*nic2* system of nicotine reduction, the *BBL* mutant system was also found to reduce cured leaf yields by up to 29%, with associated reductions in cured quality of up to 15%. The reasons for observed associations between reduced nicotine and these measures of commercial significance are unknown, but such data is evidence of complexities associated with plant biochemical and physiological processes that should not be underestimated. It is possible that modified harvesting and curing regimes may lessen the negative impact of this allelic variability on cured leaf quality in low nicotine materials. It may also be important to study the potential for increased insect herbivory on tobacco plants with reduced nicotine accumulation, as increased insect feeding has been observed on *Nicotiana* plants with silenced nicotine biosynthetic genes (Steppuhn et al., 2004).

Nolke et al. (2018) reported increases in polyamine content to be correlated with delays in maturation and senescence in a study of *nic1*/*nic2* isolines of burley tobacco, with problems being most apparent in the lowest alkaloid material. Delayed field ripening of tobacco can translate into reduced cured leaf quality. It is worthwhile to point out that the increased green leaf color frequently associated with the *nic1*/*nic2* type of genetic variation was not as obvious in the *BBL* mutant material. This may have contributed to less severe reductions in cured leaf quality for the *BBL* mutant material as compared to that observed for some of the *nic1*/*nic2* genetic stocks.

No *BBL* mutant combination exhibited percent nicotine lower than that exhibited by the *nic1*/*nic1 nic2*/*nic2* NILs evaluated in the current research. We were, however, able to combine mutations in *BBL-a*, *BBL-b*, and *BBL-c* with the recessive alleles at the *Nic1* and *Nic2* loci. Such genotypes were found, on average, to exhibit nicotine levels in the top leaves harvested 21 days after topping that were lower than those observed for triple *BBL*-mutant materials by themselves. Because these genotypes were not inbred lines, we did not carry out evaluation of this material for yield and quality.

BBL enzymes clearly play a role in nicotine biosynthesis, but their precise function is yet to be completely understood. Tobacco genotypes with deleterious mutations in the three most highly expressed *BBL* genes also exhibit severe reductions in anatabine and modest reductions in anabasine, thus indicating a role for BBL-a, BBL-b, and BBL-c enzymes in the biosynthesis of these alkaloids. Because the biosynthesis of nicotine, anatabine, and anabasine all involve condensation reactions with a nicotinic acid derivative, it is reasonable to presume that *BBL-a*, *BBL-b*, and *BBL-c* encode for enzymes involved with the activation of nicotinic acid or play a role in the completion of condensation reactions involving a pyridine ring. It was previously reported that a novel alkaloid designated as dihydrometanicotine (DMN) accumulated in the roots of tobacco plants with interrupted BBL enzyme activity (Kajikawa et al., 2011; Lewis et al., 2015). As pointed out by Kajikawa et al. (2011), the chemical structure of DMN suggests that the condensation of the *N*-methylpyrrolinium cation and pyridine rings may precede the biochemical step catalyzed by *BBL* enzymes. Although it is possible that DMN may be a substrate for BBL enzymes, its failure to be oxidized to nicotine or other known alkaloids in *in vitro* assays suggests the possibility that DMN is instead a stable derivative of the authentic, presumably unstable BBL substrate (Kajikawa et al., 2011).

There are currently no reported experiments where genetic manipulation of the tobacco plant has resulted in reductions of nicotine to non-measurable levels in field-grown plants. In research reported here, we abolished enzyme functionality for all known members of the *BBL* family for which corresponding genes can be identified in public genomic sequence databases. Likewise, use of RNA interference to dramatically reduce expression of alternative gene families involved in nicotine biosynthesis has not resulted in reductions of tobacco nicotine content to non-detectable levels (Lewis, 2018). Tobacco plants homozygous for deleterious mutations in all known nicotine demethylase genes normally expressed in the leaves or roots also similarly still accumulate appreciable amounts of nornicotine (Lewis et al., 2010). This leads to interesting speculation about the complete genetic and biochemical basis of nicotine and nornicotine production and accumulation in tobacco. The specific enzymatic or chemical steps involved in the condensation reaction between a nicotinic acid-derived intermediate and the *N*-methylpyrrolinium cation in the formation of nicotine are still not understood (Kajikawa et al., 2011). The accumulation of appreciable amounts of nicotine in the absence of any believed functional BBL enzyme activity suggests that some nicotine and its demethylated counterpart, nornicotine, may be biosynthesized via an alternative route. Heim et al. (2007) suggested the possibility of an alternative route for nornicotine biosynthesis that more directly involves putrescine and that is independent of nicotine. In the present research, we observed significantly elevated levels of nornicotine in tobacco lines possessing specific *BBL* mutant combinations, supporting the possible role of a currently uncharacterized route for biosynthesis of this alkaloid. The possible role of promiscuous activity by enzymes currently not identified to be primarily involved with nicotine biosynthesis cannot be ruled out for affecting the accumulation of the remaining nicotine in tobacco genotypes silenced for a single gene family. The observation of further reductions in nicotine when mutations in *BBL-a*, *BBL-b*, and *BBL-c* were combined with the recessive alleles at the *Nic1* and *Nic2* loci may be informative, however. This finding suggests that gene(s) controlling the accumulation of the majority of the remaining nicotine in genotypes homozygous for the *BBL* mutations are likely positively regulated by transcription factors reported to reside at the *Nic1* and *Nic2* loci (Shoji et al., 2010; Humphry et al., 2018; Pramod et al., 2019).

## Data Availability Statement

All datasets generated for this study are included in the article/Supplementary Material.

## Author Contributions

KD-S, WS, and CH performed the genotyping. TS performed the chemical analyses. RL and RD designed the research. RL analyzed the data and wrote the manuscript. All authors reviewed the manuscript.

## Conflict of Interest

NCSU has licensed specific genetic materials described in this manuscript to 22nd Century Inc.
