# Supplementary material for: Genetic and Agronomic Analysis of Tobacco Genotypes Exhibiting Reduced Nicotine Accumulation Due to Induced Mutations in Berberine Bridge Like (BBL) Genes
Source: Front Plant Sci. 2020 Apr 3;11:368. doi: 10.3389/fpls.2020.00368 (PMC7147384; doi:10.3389/fpls.2020.00368)
Supplement: Supplementary file 1 [file Table_1.pdf]

Supplementary Table 1. Genotypes evaluated for alkaloid accumulation and agronomic traits.

Line Designation	*BBL* Genotype	*Nic1* + *Nic2* Genotype
K326	*BBL-a* /*BBL-a* *BBL-b* /*BBL-b* *BBL-c* /*BBL-c* *BBL-d1* /*BBL-d1* *BBL-d2* /*BBL-d2* *BBL-e* /*BBL-e*	*Nic1* /*Nic1* *Nic2* /*Nic2*
K326 (000)	*BBL-a* /*BBL-a* *BBL-b* /*BBL-b* *BBL-c* /*BBL-c* *BBL-d1* /*BBL-d1* *BBL-d2* /*BBL-d2* *BBL-e* /*BBL-e*	*Nic1* /*Nic1* *Nic2* /*Nic2*
K326 (002)	*BBL-a* /*BBL-a* *BBL-b* /*BBL-b* *bbl-c* /*bbl-c* *BBL-d1* /*BBL-d1* *BBL-d2* /*BBL-d2* *BBL-e* /*BBL-e*	*Nic1* /*Nic1* *Nic2* /*Nic2*
K326 (020)	*BBL-a* /*BBL-a* *bbl-b* /*bbl-b* *BBL-c* /*BBL-c* *BBL-d1* /*BBL-d1* *BBL-d2* /*BBL-d2* *BBL-e* /*BBL-e*	*Nic1* /*Nic1* *Nic2* /*Nic2*
K326 (200)	*bbl-a* /*bbl-a* *BBL-b* /*BBL-b* *BBL-c* /*BBL-c* *BBL-d1* /*BBL-d1* *BBL-d2* /*BBL-d2* *BBL-e* /*BBL-e*	*Nic1* /*Nic1* *Nic2* /*Nic2*
K326 (022)	*BBL-a* /*BBL-a* *bbl-b* /*bbl-b* *bbl-c* /*bbl-c* *BBL-d1* /*BBL-d1* *BBL-d2* /*BBL-d2* *BBL-e* /*BBL-e*	*Nic1* /*Nic1* *Nic2* /*Nic2*
K326 (202)	*bbl-a* /*bbl-a* *BBL-b* /*BBL-b* *bbl-c* /*bbl-c* *BBL-d1* /*BBL-d1* *BBL-d2* /*BBL-d2* *BBL-e* /*BBL-e*	*Nic1* /*Nic1* *Nic2* /*Nic2*
K326 (220)	*bbl-a* /*bbl-a* *bbl-b* /*bbl-b* *BBL-c* /*BBL-c* *BBL-d1* /*BBL-d1* *BBL-d2* /*BBL-d2* *BBL-e* /*BBL-e*	*Nic1* /*Nic1* *Nic2* /*Nic2*
K326 (111)	*BBL-a* /*bbl-a* *BBL-b* /*bbl-b* *BBL-c* /*bbl-c* *BBL-d1* /*BBL-d1* *BBL-d2* /*BBL-d2* *BBL-e* /*BBL-e*	*Nic1* /*Nic1* *Nic2* /*Nic2*
K326 (222)	*bbl-a* /*bbl-a* *bbl-b* /*bbl-b* *bbl-c* /*bbl-c* *BBL-d1* /*BBL-d1* *BBL-d2* /*BBL-d2* *BBL-e* /*BBL-e*	*Nic1* /*Nic1* *Nic2* /*Nic2*
TN 90	*BBL-a* /*BBL-a* *BBL-b* /*BBL-b* *BBL-c* /*BBL-c* *BBL-d1* /*BBL-d1* *BBL-d2* /*BBL-d2* *BBL-e* /*BBL-e*	*Nic1* /*Nic1* *Nic2* /*Nic2*
TN 90 (000)	*BBL-a* /*BBL-a* *BBL-b* /*BBL-b* *BBL-c* /*BBL-c* *BBL-d1* /*BBL-d1* *BBL-d2* /*BBL-d2* *BBL-e* /*BBL-e*	*Nic1* /*Nic1* *Nic2* /*Nic2*
TN 90 (002)	*BBL-a* /*BBL-a* *BBL-b* /*BBL-b* *bbl-c* /*bbl-c* *BBL-d1* /*BBL-d1* *BBL-d2* /*BBL-d2* *BBL-e* /*BBL-e*	*Nic1* /*Nic1* *Nic2* /*Nic2*
TN 90 (020)	*BBL-a* /*BBL-a* *bbl-b* /*bbl-b* *BBL-c* /*BBL-c* *BBL-d1* /*BBL-d1* *BBL-d2* /*BBL-d2* *BBL-e* /*BBL-e*	*Nic1* /*Nic1* *Nic2* /*Nic2*
TN 90 (200)	*bbl-a* /*bbl-a* *BBL-b* /*BBL-b* *BBL-c* /*BBL-c* *BBL-d1* /*BBL-d1* *BBL-d2* /*BBL-d2* *BBL-e* /*BBL-e*	*Nic1* /*Nic1* *Nic2* /*Nic2*
TN 90 (022)	*BBL-a* /*BBL-a* *bbl-b* /*bbl-b* *bbl-c* /*bbl-c* *BBL-d1* /*BBL-d1* *BBL-d2* /*BBL-d2* *BBL-e* /*BBL-e*	*Nic1* /*Nic1* *Nic2* /*Nic2*
TN 90 (202)	*bbl-a* /*bbl-a* *BBL-b* /*BBL-b* *bbl-c* /*bbl-c* *BBL-d1* /*BBL-d1* *BBL-d2* /*BBL-d2* *BBL-e* /*BBL-e*	*Nic1* /*Nic1* *Nic2* /*Nic2*
TN 90 (220)	*bbl-a* /*bbl-a* *bbl-b* /*bbl-b* *BBL-c* /*BBL-c* *BBL-d1* /*BBL-d1* *BBL-d2* /*BBL-d2* *BBL-e* /*BBL-e*	*Nic1* /*Nic1* *Nic2* /*Nic2*
TN 90 (222)	*bbl-a* /*bbl-a* *bbl-b* /*bbl-b* *bbl-c* /*bbl-c* *BBL-d1* /*BBL-d1* *BBL-d2* /*BBL-d2* *BBL-e* /*BBL-e*	*Nic1* /*Nic1* *Nic2* /*Nic2*
TN 90 SRC	*BBL-a* /*BBL-a* *BBL-b* /*BBL-b* *BBL-c* /*BBL-c* *BBL-d1* /*BBL-d1* *BBL-d2* /*BBL-d2* *BBL-e* /*BBL-e*	*Nic1* /*Nic1* *Nic2* /*Nic2*
TN 90 SRC (000)	*BBL-a* /*BBL-a* *BBL-b* /*BBL-b* *BBL-c* /*BBL-c* *BBL-d1* /*BBL-d1* *BBL-d2* /*BBL-d2* *BBL-e* /*BBL-e*	*Nic1* /*Nic1* *Nic2* /*Nic2*
TN 90 SRC (002)	*BBL-a* /*BBL-a* *BBL-b* /*BBL-b* *bbl-c* /*bbl-c* *BBL-d1* /*BBL-d1* *BBL-d2* /*BBL-d2* *BBL-e* /*BBL-e*	*Nic1* /*Nic1* *Nic2* /*Nic2*
TN 90 SRC (020)	*BBL-a* /*BBL-a* *bbl-b* /*bbl-b* *BBL-c* /*BBL-c* *BBL-d1* /*BBL-d1* *BBL-d2* /*BBL-d2* *BBL-e* /*BBL-e*	*Nic1* /*Nic1* *Nic2* /*Nic2*
TN 90 SRC (200)	*bbl-a* /*bbl-a* *BBL-b* /*BBL-b* *BBL-c* /*BBL-c* *BBL-d1* /*BBL-d1* *BBL-d2* /*BBL-d2* *BBL-e* /*BBL-e*	*Nic1* /*Nic1* *Nic2* /*Nic2*
TN 90 SRC (022)	*BBL-a* /*BBL-a* *bbl-b* /*bbl-b* *bbl-c* /*bbl-c* *BBL-d1* /*BBL-d1* *BBL-d2* /*BBL-d2* *BBL-e* /*BBL-e*	*Nic1* /*Nic1* *Nic2* /*Nic2*
TN 90 SRC (202)	*bbl-a* /*bbl-a* *BBL-b* /*BBL-b* *bbl-c* /*bbl-c* *BBL-d1* /*BBL-d1* *BBL-d2* /*BBL-d2* *BBL-e* /*BBL-e*	*Nic1* /*Nic1* *Nic2* /*Nic2*
TN 90 SRC (220)	*bbl-a* /*bbl-a* *bbl-b* /*bbl-b* *BBL-c* /*BBL-c* *BBL-d1* /*BBL-d1* *BBL-d2* /*BBL-d2* *BBL-e* /*BBL-e*	*Nic1* /*Nic1* *Nic2* /*Nic2*
TN 90 SRC (222)	*bbl-a* /*bbl-a* *bbl-b* /*bbl-b* *bbl-c* /*bbl-c* *BBL-d1* /*BBL-d1* *BBL-d2* /*BBL-d2* *BBL-e* /*BBL-e*	*Nic1* /*Nic1* *Nic2* /*Nic2*

TN 90 SRC (222002)	*bbl-a /bbl-a bbl-b /bbl-b bbl-c /bbl-c BBL-d1 /BBL-d1 BBL-d2 /BBL-d2 bbl-e /bbl-e*	*Nic1 /Nic1 Nic2 /Nic2*
TN 90 SRC (222020)	*bbl-a /bbl-a bbl-b /bbl-b bbl-c /bbl-c BBL-d1 /BBL-d1 bbl-d2 /bbl-d2 BBL-e /BBL-e*	*Nic1 /Nic1 Nic2 /Nic2*
TN 90 SRC (222200)	*bbl-a /bbl-a bbl-b /bbl-b bbl-c /bbl-c bbl-d1 /bbl-d1 BBL-d2 /BBL-d2 BBL-e /BBL-e*	*Nic1 /Nic1 Nic2 /Nic2*
TN 90 SRC (222022)	*bbl-a /bbl-a bbl-b /bbl-b bbl-c /bbl-c BBL-d1 /BBL-d1 bbl-d2 /bbl-d2 bbl-e /bbl-e*	*Nic1 /Nic1 Nic2 /Nic2*
TN 90 SRC (222202)	*bbl-a /bbl-a bbl-b /bbl-b bbl-c /bbl-c bbl-d1 /bbl-d1 BBL-d2 /BBL-d2 bbl-e /bbl-e*	*Nic1 /Nic1 Nic2 /Nic2*
TN 90 SRC (222220)	*bbl-a /bbl-a bbl-b /bbl-b bbl-c /bbl-c bbl-d1 /bbl-d1 bbl-d2 /bbl-d2 BBL-e /BBL-e*	*Nic1 /Nic1 Nic2 /Nic2*
TN 90 SRC (222222)	*bbl-a /bbl-a bbl-b /bbl-b bbl-c /bbl-c bbl-d1 /bbl-d1 bbl-d2 /bbl-d2 bbl-e /bbl-e*	*Nic1 /Nic1 Nic2 /Nic2*
NC95	*BBL-a /BBL-a BBL-b /BBL-b BBL-c /BBL-c BBL-d1 /BBL-d1 BBL-d2 /BBL-d2 BBL-e /BBL-e*	*Nic1 /Nic1 Nic2 /Nic2*
MAFC5	*BBL-a /BBL-a BBL-b /BBL-b BBL-c /BBL-c BBL-d1 /BBL-d1 BBL-d2 /BBL-d2 BBL-e /BBL-e*	*Nic1 /Nic1 nic2 /nic2*
LMAFC34	*BBL-a /BBL-a BBL-b /BBL-b BBL-c /BBL-c BBL-d1 /BBL-d1 BBL-d2 /BBL-d2 BBL-e /BBL-e*	*nic1 /nic1 Nic2 /Nic2*
LAFC53	*BBL-a /BBL-a BBL-b /BBL-b BBL-c /BBL-c BBL-d1 /BBL-d1 BBL-d2 /BBL-d2 BBL-e /BBL-e*	*nic1 /nic1 nic2 /nic2*
Burley 21	*BBL-a /BBL-a BBL-b /BBL-b BBL-c /BBL-c BBL-d1 /BBL-d1 BBL-d2 /BBL-d2 BBL-e /BBL-e*	*Nic1 /Nic1 Nic2 /Nic2*
HI Burley 21	*BBL-a /BBL-a BBL-b /BBL-b BBL-c /BBL-c BBL-d1 /BBL-d1 BBL-d2 /BBL-d2 BBL-e /BBL-e*	*Nic1 /Nic1 nic2 /nic2*
LI Burley 21	*BBL-a /BBL-a BBL-b /BBL-b BBL-c /BBL-c BBL-d1 /BBL-d1 BBL-d2 /BBL-d2 BBL-e /BBL-e*	*nic1 /nic1 Nic2 /Nic2*
LA Burley 21	*BBL-a /BBL-a BBL-b /BBL-b BBL-c /BBL-c BBL-d1 /BBL-d1 BBL-d2 /BBL-d2 BBL-e /BBL-e*	*nic1 /nic1 nic2 /nic2*
